# Novel Cyclized Hexapeptide‐9 Outperforms Retinol Against Skin Aging: A Randomized, Double‐Blinded, Active‐ and Vehicle‐Controlled Clinical Trial

**DOI:** 10.1111/jocd.70290

**Published:** 2025-06-30

**Authors:** Huailong Chang, Kan Tao, Yuge Yang, Yanling Wang, Mengru Ge, Xiaoli Wang, Shengnan Tang, Haining Yu

**Affiliations:** ^1^ Global R&D Center Shanghai Chicmax Cosmetic Co. Ltd. Shanghai China; ^2^ Shanghai Kans Biotech Co. Ltd. Shanghai China

**Keywords:** clinical trial, cyclized hexapeptide‐9, efficacy evaluation, retinol, skin aging, wrinkles

## Abstract

**Background:**

The functionality and regenerative capacity of skin progressively deteriorate with aging. Topical use of peptides with a hyper‐safety profile has been implicated in replacing retinol for skin anti‐aging use, but is limited due to low stability and poor skin permeability.

**Aims:**

In this randomized, double‐blinded, active‐ and vehicle‐controlled clinical trial, we aim to evaluate the efficacy of an innovative cyclized hexapeptide‐9 (CHP‐9) with increased stability and skin permeability on skin aging compared to retinol.

**Methods:**

Healthy volunteers with aging skin were randomly assigned to twice‐daily topical use of 0.002% CHP‐9 serum, 0.002% retinol serum, or vehicle serum for 56 days.

**Results:**

CHP‐9 treatment significantly decreased the number, area, and roughness of both crow's feet (−2.20, 95% CI: −4.38, −0.03; −3.95, 95% CI: −5.80, −2.11; −1.95, 95% CI: −3.30, −0.59, respectively) and forehead wrinkles (−2.88, 95% CI: −4.21, −1.56; −4.90, 95% CI: −5.97, −3.82; −3.96, 95% CI: −5.92, −2.01, respectively), while retinol only decreased the area of crow's feet (−2.23, 95% CI: −3.86, −0.60) and the number and area of forehead wrinkles (−1.05, 95% CI:‐1.69, −0.41). Except for the roughness of crow's feet, CHP‐9 demonstrated significantly larger extent of effects than retinol did on all other outcomes. Furthermore, long‐term use of CHP‐9 showed time‐dependent augmentation in its potency to reduce the number of crow's feet, and the number, area, and roughness of forehead wrinkles.

**Conclusions:**

In conclusion, CHP‐9 is more potent than retinol in improving skin aging‐related symptoms, especially for long‐term use. Cyclization of collagen peptides may present a preventive/therapeutic option for skin aging.

## 1 Introduction

Skin aging is a multifactorial process driven by both intrinsic and extrinsic factors, characterized by a progressive decline in its functionality and regenerative capacity. Intrinsic aging is marked by loss of elasticity, dry skin, and fine lines, while extrinsic aging is manifested by deeper wrinkles, uneven pigmentation, and telangiectasias [1, 2]. Together, intrinsic and extrinsic aging damage the structural integrity and functional capacity of all layers of the skin. Aging of the epidermis is associated with a significant decrease in the thickness by 10%–50% between the ages of 30 and 80 years old [3, 4]. The connections between the epidermis and the dermis are also gradually degraded, resulting in reduced blood supply to the epidermis. Moreover, skin aging is also associated with reduced levels and disorganization of key components of extracellular matrix (ECM) that support the normal functions of skin cells, including hyaluronic acid, collagens, and elastin fibers. These result in the increased dryness of skin, loss of skin elasticity, formation of wrinkles, and slower regeneration from skin damage [5].

Retinol has been widely used topically to reduce wrinkles and has been approved by the Food and Drug Administration (FDA) for anti‐aging therapies since 1996 [6]. The anti‐aging effects of retinoids have been associated with the promotion of cell proliferation and synthesis of collagen and elastin fibers, and the inhibition of collagen degradation [7, 8, 9, 10, 11]. However, the clinical application of retinoids has been limited by their poor water solubility, photo‐instability, and high irritating potency, leading to the development of novel derivatives and/or drug delivery technologies to increase the stability while decreasing the irritating potential [12, 13, 14, 15]. As a promising substitution for retinoids, peptides have drawn much attention due to their broad spectrum of physiological properties, including antioxidation, anti‐aging, moisturizing, collagen‐stimulating, and wound‐healing [16, 17, 18], and, in particular, their hyper‐safety and hypo‐allergenicity profile [19]. However, conventional peptides are linear and flexible in structure, with amino and carboxyl groups located at either end, making them not only polar in nature but also prone to being degraded by proteolysis. Interestingly, pharmacological studies have suggested that cyclization of linear peptides by linking the two ends with a covalent bond could increase their conformational rigidity, which could in turn enhance their stability, membrane permeability, and target selectivity [20, 21]. However, little has been done to study the effects of cyclization on the therapeutic potential of the topical use of peptides. To date, only one cyclotetrapeptide‐24 aminocyclohexane carboxylate has ever been introduced for cosmetic use, claiming to improve skin elasticity and reduce wrinkles with enhanced binding to integrins. However, no published study has confirmed these effects in a clinical trial context.

As a collagen peptide, hexapeptide‐9 (Gly‐Pro‐Gln‐Gly‐Pro‐Gln) has been designed to provide curative and/or preventive treatment for skin aging and to enhance skin appearance [22]. We have previously synthesized a cyclohexapeptide‐9 (CHP‐9) by linking the head and tail of hexapeptide‐9 with an amide bond and have confirmed its superior safety. In the present study, we aim to evaluate the anti‐aging effects of CHP‐9 in a clinical trial set by comparing it to retinol, the gold standard of anti‐aging treatment. To our best knowledge, this is the first study on the effects of a cyclic collagen peptide in comparison to retinol, in terms of anti‐aging efficacy in human subjects.

## 2 Materials and Methods

### 2.1 Trial Design

This was a three‐arm, randomized, double‐blinded, and vehicle‐controlled study conducted in mainland China. Written informed consent was obtained from each patient after the procedures, risks, and benefits had been explained. The trial protocol was approved by the Institutional Review Board and Ethics Committee in the study center (Approval No.: 2024‐QX‐05‐02). The trial was conducted in accordance with the principles of the Declaration of Helsinki and Good Clinical Practice guidelines of the International Council for Harmonization.

### 2.2 Participants

Healthy adult male and female participants aged between 30 and 55 years were recruited in the current study. All the participants were screened for mildly to moderately damaged skin barrier function, which was prone to erythema with a TEWL ≥ 15 g/h/m^2^. In addition, the facial skin of the participants was relatively dull with lines and wrinkles in the face and corner of the eye. The forehead wrinkles and crow's feet were graded by experienced technicians by comparing them to guideline photographs, and only participants with forehead wrinkles graded ≥ 2 and crow's feet graded ≥ 3 were recruited. Participants were deemed ineligible if they had used topical retinoids, α‐hydroxy acids, salicylic acid, or hydroquinone within 3 months before the study.

### 2.3 Treatments

Participants were randomized to receive a 0.002% (*w*/*w*) CHP‐9 serum, a 0.002% (*w*/*w*) retinol serum, or the matching vehicle control using a computer‐generated randomization code (Table S1). Both the investigators and participants were blinded to the treatment that the participants applied. All the participants were instructed to apply the serum to their entire face, excluding eyes, mouth, and nostrils, twice daily for 56 days. No other skincare products, medications, or supplements that may have impacts on the study results were allowed during the study.

### 2.4 Efficacy Evaluations

Evaluations were conducted at baseline and after 14, 28, and 56 days of treatment. All the evaluations were conducted under an ambient temperature and relative humidity of 21°C ± 1°C and 50% ± 10% relative humidity, respectively. Participants were instructed to clean their faces with a mild cleanser and acclimate for 30 min before the evaluation. The primary efficacy outcome measures were crow's feet and forehead wrinkling, including instrumental measurements of their numbers, area, and surface roughness. A PRIMOS^CR^ (Canfield Scientific, Parsippany, NJ) system was employed to take photos of the faces of all the participants, based on which the number, area, and surface roughness of crow's feet and forehead wrinkles were further quantified by the associated software. Secondary efficacy outcome measures included instrumental measurements of skin elasticity and firmness, skin pigmentation, and barrier functions. In this case, a VISIA‐CR (Canfield Scientific, Parsippany, NJ) system was first adopted to capture images of the participants' faces and quantify the percentage of red area relative to the whole area of the face. Then, a Cutometer dual MPA 580 (Courage + Khazaka electronic GmbH, Köln, Germany) system was used to measure the R2, R5, and R7 values reflecting skin elasticity, and the F4 value for skin firmness, respectively. In addition, other parameters were measured by connecting specific probes to the system, including Ultrascan UC22 for the thickness and density of the epidermis, Corneometer CM 825 for the water content in the stratum corneum, Tewameter TM Hex for the TEWL, Mexameter MX 18 for the melanin and hemoglobin content, Colorimeter CL 400 for the *Lab* values and ITA, and Skin‐Glossymeter GL 200 for skin glossiness, respectively.

Other secondary efficacy outcome measures were dermatological assessment of skin wrinkling, pigmentation, firmness, and barrier function. For the assessment of dryness, roughness, firmness, evenness, dullness, glossiness, and erythema, a 10‐point scale was adopted. Fine lines in the cheek and undereye wrinkles were also assessed using a 10‐point scale, while crow's feet, forehead wrinkles, and nasolabial folds were assessed with a 7‐point, a 9‐point, and an 8‐point scale, respectively. For all of the scales, a higher score indicates a worse appearance of the skin. All those scales were customized according to the Skin Atlas Volume 2: Asian Type and have been validated for accuracy.

### 2.5 Safety

Participants were monitored for signs and symptoms of local irritation (erythema, infiltration, papules, blisters, edema, and bullae) at every visit, and the intensity of the reaction was rated with a 5‐point scale, with a higher score indicating a more severe reaction. Other adverse events that were spontaneously reported or noticed by the investigators were also recorded at each visit. Participants were also asked to assess their overall tolerability at each visit.

### 2.6 Statistical Analysis

Participants who were randomized and treated were included in the safety analysis. Participants who were treated and finished all the measurements and assessments were included in all the demographic and efficacy analyses. Assuming a type I error rate of 0.05 (2‐sided), an a priori power analysis indicated that a sample size of 17 participants per group would provide a 90% power to detect a 120% between‐group difference in the area of crow's feet. One previous study has indicated that formulations containing hexapeptide‐9 may reduce the area of crow's feet by 130%–189% [23]. To assess the effects of retinol and CHP‐9 versus placebo on indicators of skin aging, we used generalized estimating equation (GEE) models with an exchangeable working correlation structure and an identity link function. The GEE models account for the within‐subject correlation for data with repeated measures over time and provide marginal estimates. Dermatological assessed outcomes were analyzed as ordinal multinominal variables with a GEE solver for correlated nominal or ordinal multinomial responses using a local odds ratios parameterization [24, 25]. Model predictors included a treatment indicator, time of the visit, baselines, and a treatment‐by‐time interaction term. To account for the impacts of the baseline on the absolute values of the measured parameters, a percentage of change relative to the baseline was calculated for each parameter at each visit. In addition, multiple comparisons were performed between different groups at different time points using a resampling‐based method to adjust the *p* values to account for the type I error rate. All analyses were performed using R (version 4.4.0), in association with packages *longpower*, *geepack*, *geeasy*, *multgee*, and *multtest*. All the tests were 2‐sided with 0.05 significance levels.

## 3 Results

### 3.1 Characteristics of Participants

Ninety‐six Asian participants were enrolled and randomized at a ratio of 1:1:1 to receive one of the three treatments: a vehicle control serum or a serum supplemented with either 0.002% (*w*/*w*) retinol or 0.002% (*w*/*w*) CHP‐9. Of the 96 randomized to treatments, 91 (94.8%) completed the study (Figure S1). Five participants dropped out due to nonattendance at study visits. Baseline demographics were comparable among the groups (Table 1).

**TABLE 1:** Participants' demographics and skin conditions at baseline.

Demographics and skin conditions	Treatment group		
	Control	Retinol	CHP‐9
No.	30	31	30
Sex, *n* (%)			
Female	29 (96.7)	29 (93.5)	30 (100.0)
Male	1 (3.3)	2 (6.5)	0 (0.0)
Age, mean ± SD, years	44.4 ± 5.8	41.5 ± 5.5	44.9 ± 7.2
Crow's feet score, *n* (%)			
2	0 (0.0)	1 (3.2)	0 (0.0)
3	7 (23.3)	15 (48.4)	9 (30.0)
4	14 (46.7)	12 (38.7)	11 (36.7)
5	7 (23.3)	2 (6.5)	5 (16.7)
6	2 (6.7)	1 (3.2)	5 (16.7)
Forehead wrinkles score, *n* (%)			
2	16 (53.3)	20 (64.5)	11 (36.7)
3	7 (23.3)	4 (12.9)	7 (23.3)
4	4 (13.3)	6 (19.4)	8 (26.7)
5	3 (10.0)	0 (0.0)	4 (13.3)
6	0 (0.0)	1 (3.2)	0 (0.0)

### 3.2 Primary Outcomes

#### 3.2.1 Crow's Feet

**FIGURE 1:**
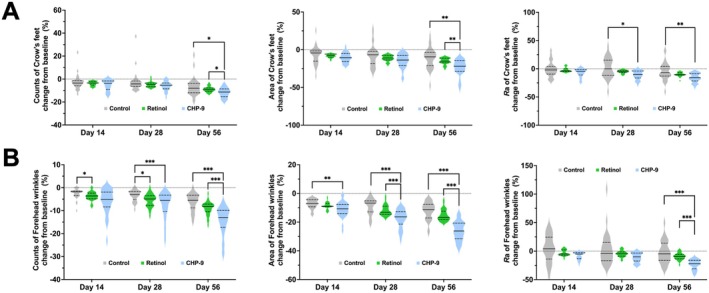
Relative changes from baseline on days 14, 28, and 56 for (A) number, area, and roughness of crow's feet, and (B) number, area, and roughness of forehead wrinkles. Relative change for each parameter was calculated by dividing the change from baseline by the baseline. Multiple comparisons were performed using a resampling‐based method to adjust the *P* values to account for type I error rate. The solid line in the middle of the violin plot represents the median, and the lower and upper dashed lines represent the 25th and 75th quantiles, respectively. *p ≤ 0.05, **p ≤ 0.01, and ***p ≤ 0.001. CHP‐9, cyclohexapeptide‐9.

The number of crow's feet declined significantly over time, irrespective of the treatment regimen (Figures 1A and S2). However, the extents of decrease differed significantly among treatment groups. Compared to the control group, a significant decrease of −2.20 (95% CI: −4.38, −0.03; *p* = 0.047) in the mean number of crow's feet was introduced by CHP‐9 treatment, while only −1.04 (95% CI: −2.68, 0.59; *p* = 0.212) by retinol treatment (Table 2). Pairwise comparisons of the percent changes indicated that the relative decrease in the number of crow's feet from baseline in the CHP‐9 treated group was significantly larger than that either in the control or retinol treated group at Day 56 (Figure 1A). In terms of the area of crow's feet, not only the main effects but also an interaction of treatment by time was detected for both retinol and CHP‐9, indicating the decrease in the area of crow's feet by CHP‐9 or retinol treatment varied by time. Interestingly, the decreases by retinol and CHP‐9 both progressively augmented over time, as indicated by that the difference between Day 56 and Day 28 was larger than the difference between Day 28 and Day 14 (Figure 1A). Specifically, the decrease in the area of crow's feet at Day 14 was only −1.00 mm^2^ and − 1.25 mm^2^ in the retinol and CHP‐9 treated groups, respectively, which further increased to −1.41 mm^2^ (95% CI: −2.70, −0.11; *p* = 0.033) and − 2.15 mm^2^ (95% CI: −3.57, −0.73; *p* = 0.003) on Day 28, respectively (Table 2). At Day 56, treatment with retinol and CHP‐9 decreased the area even further by −2.23 mm^2^ (95% CI: −3.86, −0.60; *p* = 0.007) and − 3.95 mm^2^ (95% CI: −5.79, −2.11; *p* < 0.001), respectively (Table 2). Pairwise comparisons indicated that, at Day 56, the differences in the relative change of the area of crow's feet between the CHP‐9 and the retinol treated groups reached statistical significance (Figure 1A). In addition to the number and area of crow's feet, CHP‐9 treatment also significantly decreased the average roughness (*R*a) by −1.95 μm (95% CI: −3.30, −0.59; *p* = 0.005), while no significant decrease was observed in the retinol treated group (Table 2). The pairwise comparison of relative changes also showed that treatment with CHP‐9 had significantly higher relative changes than the control group did at Day 28 and 56 (Figure 1A).

**TABLE 2:** GEE outputs for the primary outcomes.

Outcome	Treatment^a^	Main effects (95% CI)	Effects by time (95% CI)		
			Day 14	Day 28	Day 56
Crow's feet count	Retinol	−1.04 (−2.68, 0.59)	/	/	/
	CHP‐9	−2.20 (−4.38, −0.03)*	/	/	/
Crow's feet area (mm^2^)	**Retinol**	/	−1.00 (−2.26, 0.26)	−1.41 (−2.70, −0.11)*	−2.23 (−3.86, −0.60)**
	**CHP‐9**	/	−1.25 (−2.63, 0.13)	−2.15 (−3.57, −0.73)**	−3.95 (−5.80, −2.11)***
Crow's feet roughness (μm)	Retinol	−0.36 (−1.70, 0.98)	/	/	/
	CHP‐9	−1.95 (−3.30, −0.59)**	/	/	/
Forehead wrinkle No.	Retinol	−1.05 (−1.69, −0.41)**	/	/	/
	**CHP‐9**	/	−1.48 (−2.53, −0.43)**	−1.95 (−2.97, −0.93)***	−2.88 (−4.21, −1.56)***
Forehead wrinkle area (mm^2^)	Retinol	−0.86 (−1.44, −0.28)**	/	/	/
	**CHP‐9**	/	−1.66 (−2.35, −0.96)***	−2.74 (−3.49, −1.98)***	−4.90 (−5.97, −3.82)***
Forehead wrinkle roughness (μm)	Retinol	0.21 (−1.82, 2.25)	/	/	/
	**CHP‐9**	/	−0.96 (−3.07, 1.15)	−1.96 (−3.89, −0.04)*	−3.96 (−5.92, −2.01)***
R2	Retinol	0.02 (0.01, 0.03)**	/	/	/
	**CHP‐9**	/	0.03 (0.02, 0.05)***	0.04 (0.03, 0.05)***	0.05 (0.03, 0.07)***
R5	Retinol	−0.01 (−0.03, 0.01)	/	/	/
	**CHP‐9**	/	0.04 (0.02, 0.05)***	0.05 (0.03, 0.06)***	0.07 (0.04, 0.10)***
R7	Retinol	0.002 (−0.01, 0.01)	/	/	/
	CHP‐9	0.02 (0.01, 0.03)***	/	/	/
F4	**Retinol**	/	0.13 (−0.01, 0.28)	0.09 (−0.06, 0.23)	−0.01 (−0.19, 0.17)
	**CHP‐9**	/	0.03 (−0.12, 0.17)	−0.15 (−0.28, −0.01)*	−0.50 (−0.71, −0.29)***
b* value	Retinol	−0.24 (−0.53, 0.04)	/	/	/
	CHP‐9	−0.63 (−0.98, −0.29)***	/	/	/
L* value	Retinol	0.70 (0.27, 1.13)**	/	/	/
	**CHP‐9**		1.83 (1.48, 2.18)***	2.40 (2.07, 2.74)***	3.55 (2.60, 4.50)***
ITA°	Retinol	0.60 (−0.43, 1.64)	/	/	/
	**CHP‐9**		0.98 (−0.08, 2.05)	2.03 (1.28, 2.78)***	4.13 (2.75, 5.51)***
MI	Retinol	−3.04 (−4.19, −1.88)***	/	/	/
	**CHP‐9**	/	−5.61 (−7.38, −3.83)***	−10.49 (−12.05, −8.94)***	−20.27 (−23.21, −17.32)***
Glossiness	Retinol	−0.01 (−0.20, 0.19)	/	/	/
	**CHP‐9**	/	0.26 (0.04, 0.48)*	0.45 (0.22, 0.68)***	0.83 (0.41, 1.25)***
a* value	Retinol	−0.27 (−0.41, −0.13)***	/	/	/
	**CHP‐9**	/	−0.43 (−0.67, −0.19)***	−0.63 (−0.82, −0.444)***	−1.03 (−1.49, −0.58)***
EI	Retinol	−5.97 (−8.67, −3.27)***	/	/	/
	**CHP‐9**	/	−8.06 (−12.73, −3.39)***	−13.79 (−18.46, −9.12)***	−25.25 (−35.34, −15.15)***
Red area (mm^2^)	Retinol	−1.81 (−4.54, 0.91)	/	/	/
	CHP‐9	−3.58 (−6.15, −1.01)**	/	/	/
Epidermal thickness (μm)	Retinol	2.00 (−0.05, 4.05)	/	/	/
	**CHP‐9**	/	2.64 (−0.11, 5.39)	4.72 (2.22, 7.21)***	8.87 (4.54, 13.20)***
Epidermal density (%)	Retinol	1.57 (0.60, 2.54)**	/	/	/
	**CHP‐9**	/	1.14 (−0.41, 2.69)	2.63 (1.29, 3.96)***	5.61 (4.00, 7.21)***
Hydration (c.u.)	**Retinol**	/	1.89 (1.44, 2.35)***	2.67 (2.18, 3.16)***	4.22 (3.44, 5.00)***
	**CHP‐9**	/	3.88 (3.28, 4.48)***	5.73 (5.09, 6.38)***	9.44 (8.35, 10.53)***
TEWL (g/h/m^2^)	**Retinol**	/	−0.28 (−0.54, −0.02)*	−0.72 (−0.96, −0.48)***	−1.59 (−1.93, −1.24)***
	**CHP‐9**	/	−2.28 (−2.70, −1.86)***	−2.83 (−3.25, −2.40)***	−3.93 (−4.59, −3.27)***

*Note:* Instrumentally measured outcomes were treated as continuous variables and were analyzed with GEE models with an exchangeable working correlation structure and an identity link function. The vehicle control group was set as the reference.

Abbreviations: CHP‐9, cyclohexapeptide‐9; EI, erythema index; GEE, generalized estimating equation; ITA, individual topology angle; MI, melanin index; TEWL, trans‐epidermal water loss.

^a^ Bold text means a significant treatment‐by‐time interaction was detected.

* *p* ≤ 0.05 compared to control group.

** *p* ≤ 0.01 compared to control group.

*** *p* ≤ 0.001 compared to control group.

#### 3.2.2 Forehead Wrinkles

Treatment with retinol significantly reduced both the number and the area of forehead wrinkles by −1.05 (95% CI: −1.69, −0.41; *p* = 0.001), and − 0.86 mm^2^ (95% CI: −1.44, −0.28; *p* = 0.004), respectively (Table 2 and Figure S3). However, no significant decrease in the average roughness of forehead wrinkles was demonstrated by retinol treatment (Table 2). On the other hand, treating with CHP‐9 showed progressively augmented effects of reducing the number, area, and surface roughness of forehead wrinkles as the time of application increased (Figure 1B and S3). Compared to the control group, the decreases in the number, area, and roughness of forehead wrinkles reached −2.88 (95% CI: −4.21, −1.56; *p* < 0.001), −4.90 mm^2^ (95% CI: −5.97, −3.82; *p* < 0.001), and − 3.96 μm (95% CI: −5.92, −2.01; *p* < 0.001) on Day 56, respectively (Table 2). Multiple comparisons indicated that the improving rate for the number, area, and surface roughness was all significantly higher in the CHP‐9 group compared to the retinol group at Day 56 (Figure 1B).

### 3.3 Secondary Outcomes

#### 3.3.1 Skin Elasticity and Firmness

**FIGURE 2:**
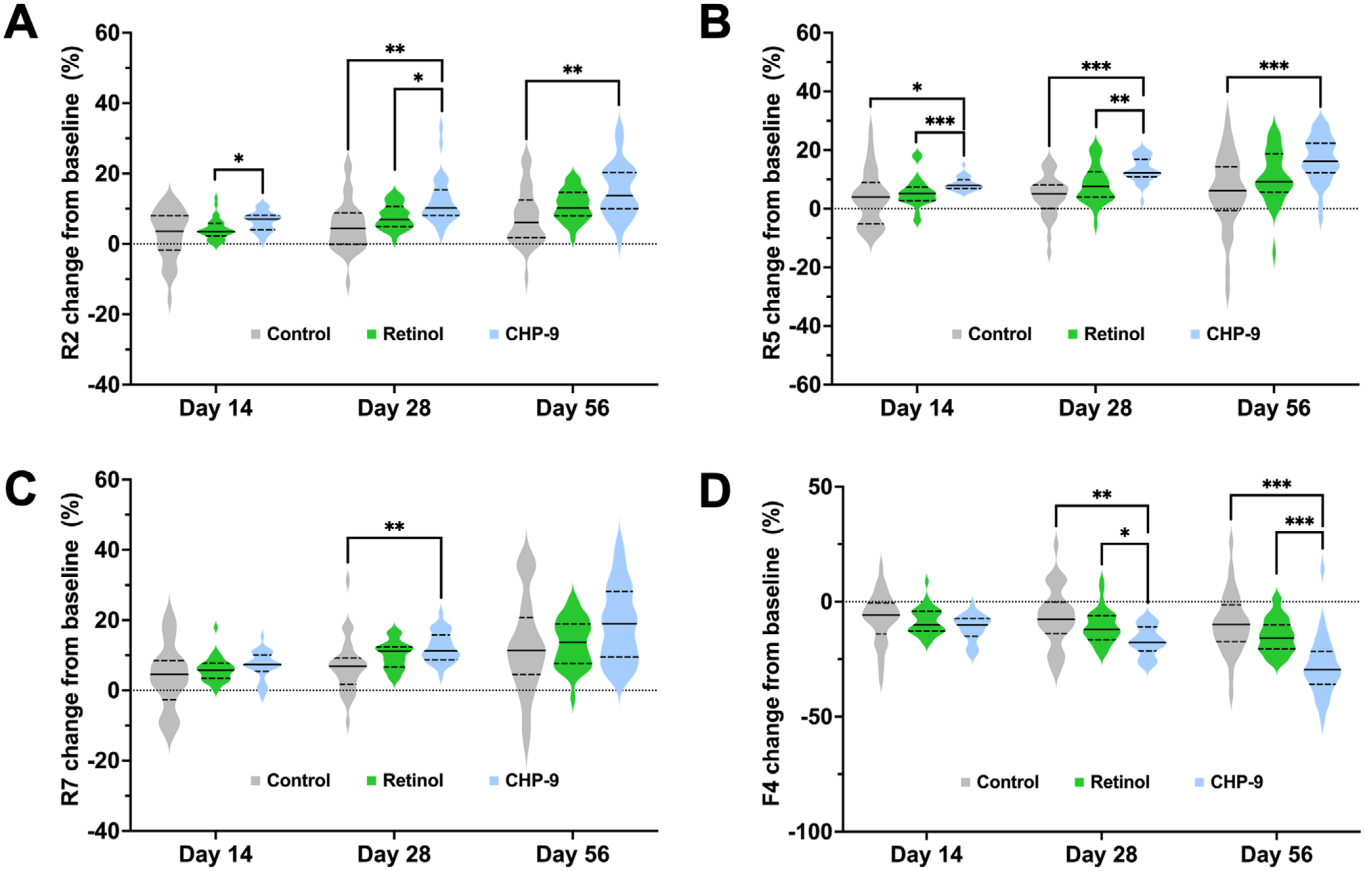
Relative changes from baseline on Days 14, 28, and 56 for (A) R2, (B) R5, (C) R7, and (D) F4 values. Relative change for each parameter was calculated by dividing the change from baseline by the baseline. Multiple comparisons were performed using a resampling‐based method to adjust the *p* values to account for type I error rate. The solid line in the middle of the violin plot represents the median, and the lower and upper dashed lines represent the 25th and 75th quantiles, respectively. *p ≤ 0.05, **p ≤ 0.01, and ***p ≤ 0.001. CHP‐9, cyclohexapeptide‐9.

Treatment with retinol increased R2 by 0.02 (95% CI: 0.01, 0.03; *p* = 0.003) compared to the control group. However, no significant increase was documented for R5 or R7 by retinol treatment (Table 2). In contrast, treatment with CHP‐9 increased R7 significantly by 0.02 (95% CI: 0.01, 0.03; *p* < 0.001) compared to the control group (Table 2). Furthermore, significant interactions of CHP‐9 treatment by time were demonstrated for both R2 and R5, in which the promoting effects on R2 and R5 progressively augmented as time increased (Table 2). On Day 56, there was a significant increase in both R2 and R5 by 0.05 (95% CI: 0.03, 0.07; *p* < 0.001) and 0.07 (95% CI: 0.04, 0.10; *p* < 0.001), respectively (Table 2). Multiple comparisons of the relative changes demonstrated that CHP‐9 improved R2 and R5 significantly more potently than retinol did at Days 14 and 28 (Figure 2A,B). However, this difference diminished on Day 56, though the relative change in the CHP‐9 group was still significantly higher than in the control group, while no significant difference was documented in the retinol group (Figure 2A,B). There was also a significant difference in the relative change of R7 between the CHP‐9 group and the control group on Day 28 (Figure 2C). In terms of skin firmness, both retinol and CHP‐9 treatment showed significant interactions with time, even though no significant change between the retinol and control group was noticed at any of the visits (Table 2). CHP‐9 treatment, however, decreased the F4 value significantly by −0.14911 mm*s (95% CI: −0.28336, −0.0148; *p* = 0.03) on Day 28, which further increased to −0.501 mm*s (95% CI: −0.715, −0.288; *p* < 0.001) on Day 56 (Table 2). Multiple comparisons indicated that CHP‐9 had significantly higher relative improvement than retinol did at Day 28 and 56. Moreover, the difference in the absolute value of F4 between the CHP‐9 and retinol groups also reached statistical significance on Day 56 (Figure 2D).

#### 3.3.2 Skin Pigmentation

**FIGURE 3:**
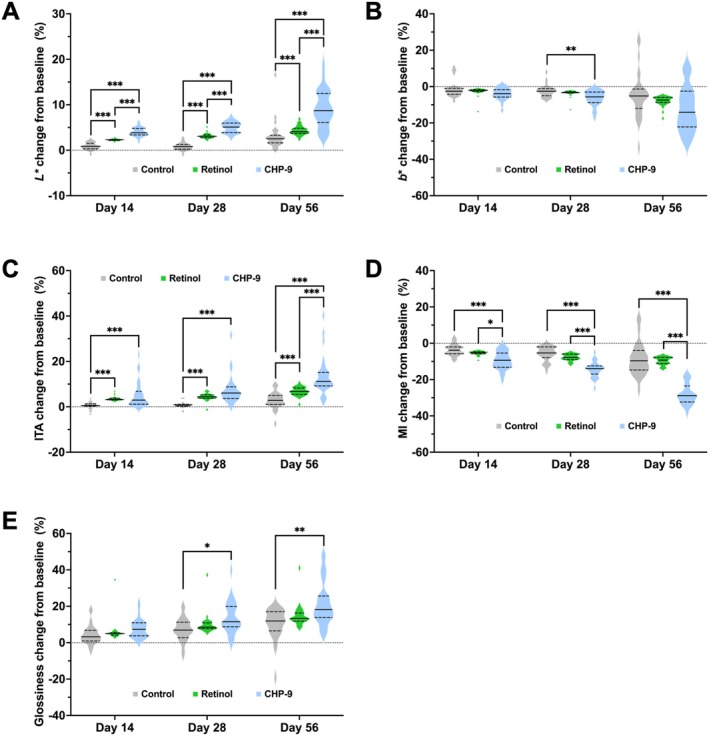
Relative changes from baseline on Days 14, 28, and 56 for (A) L* value, (B) b* value, (C) individual topology angle, (D) melanin index, and (E) skin glossiness. Relative change for each parameter was calculated by dividing the change from baseline by the baseline. Multiple comparisons were performed using a resampling‐based method to adjust the *p* values to account for type I error rate. The solid line in the middle of the violin plot represents the median, and the lower and upper dashed lines represent the 25th and 75th quantiles, respectively. *p ≤ 0.05, **p ≤ 0.01, and ***p ≤ 0.001. CHP‐9, cyclohexapeptide‐9.

The skin color of the participants was quantified using the international commission on illumination (CIE) LAB values, in which L*, a*, and b* values stand for skin luminance, red/green component, and yellow/blue component, respectively. Compared to the control, retinol treatment elicited positive effects on skin lightness by an increase in the L* value of 0.70 (95% CI: 0.27, 1.13; *p* = 0.0013; Table 2). On the other hand, CHP‐9 not only increased the L* value by 3.55 (95% CI: 2.60, 4.50; *p* < 0.001) on Day 56, but also revealed a pattern of progressively enhanced potency across the trial course (Table 2). Data from the follow‐up visits showed that the relative increases in the L* value were significantly higher in the CHP‐9 group than in the retinol group (Figure 3A). In terms of the b* value, retinol treatment showed no improvement compared to control, while CHP‐9 treatment reduced the b* value by −0.63 (95% CI: −0.98, −0.29; *p* < 0.001; Table 2) and the difference in the relative change was significant on Day 28 (Figure 3B). Consistent with no effects on the b* value, retinol also showed no significant effects on the individual topology angle (ITA), whereas CHP‐9 once again demonstrated time‐augmented effects. On Day 56, the increase in the ITA by CHP‐9 treatment reached 4.13° (95% CI: 2.75, 5.51; *p* < 0.001; Table 2), and the relative change was significantly higher than in the retinol group (Figure 3C). Moreover, this pattern of time‐enhanced potency of CHP‐9 was also observed over the melanin index (MI) and skin glossiness. The decrease in the MI and the improvement of skin glossiness progressively enhanced and reached the maximum extent on Day 56, with a value of −20.27 (95% CI: −23.21, −17.32; *p* < 0.001) and 0.83 (95% CI: 0.41, 1.25; *p* < 0.001), respectively (Table 2). On the other hand, retinol only decreased the MI by −3.04 (95% CI: −4.19, −1.88; *p* < 0.001), and induced no improvement in the skin glossiness (Table 2). Pairwise comparisons indicated that the relative changes in the MI were significantly higher in the CHP‐9 group than in the retinol group across the study (Figure 3D). There was also a significantly higher relative increase in the skin glossiness in the CHP‐9 group compared to the control group on Days 28 and 56 (Figure 3E).

#### 3.3.3 Skin Barrier and Function

**FIGURE 4:**
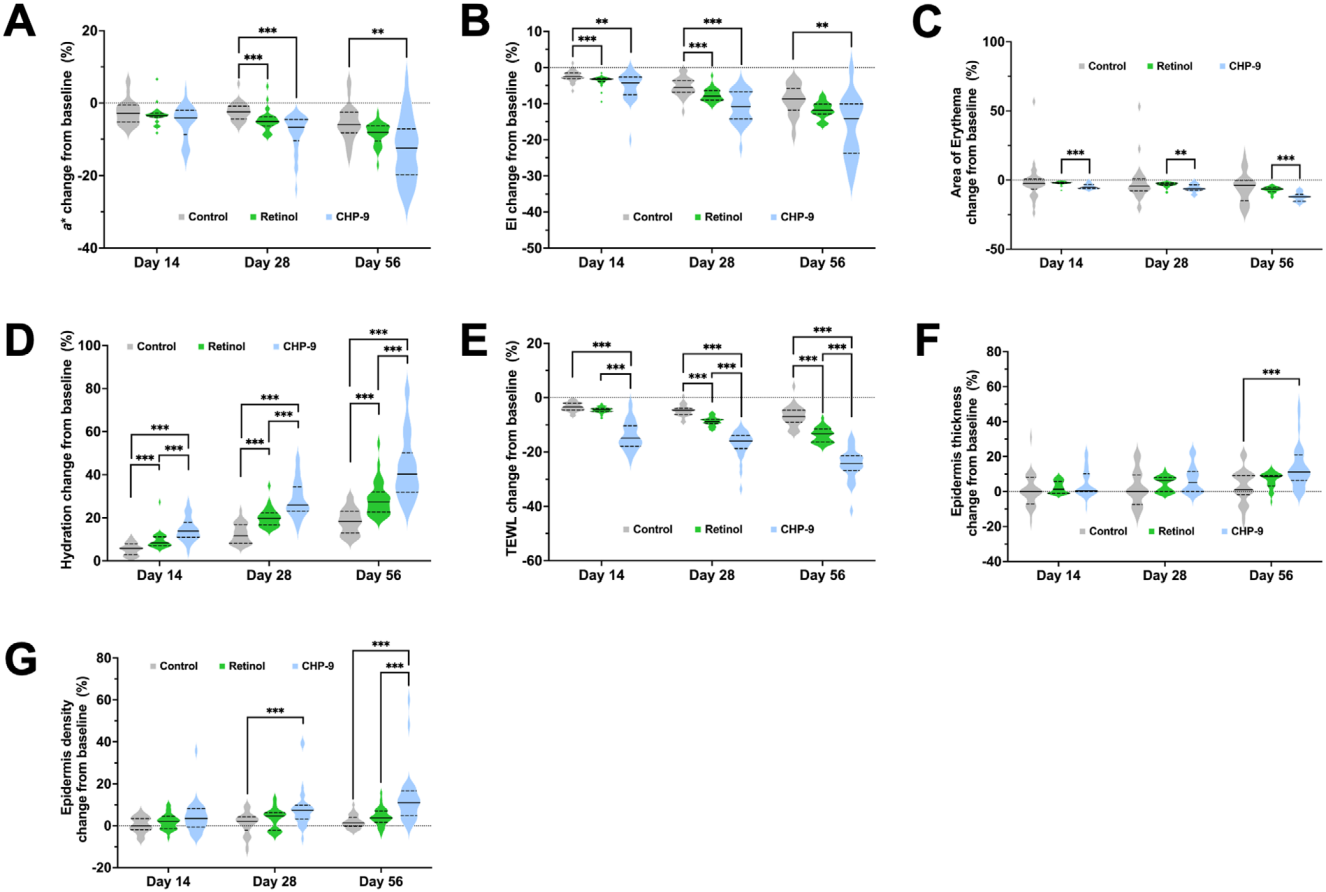
Relative changes from baseline on Days 14, 28, and 56 for (A) a* value, (B) erythema index, (C) area of erythema skin, (D) skin hydration, (E) TEWL, (F) epidermal thickness, and (G) epidermal density. Relative change for each parameter was calculated by dividing the change from baseline by the baseline. Multiple comparisons were performed using a resampling‐based method to adjust the *p* values to account for type I error rate. The solid line in the middle of the violin plot represents the median, and the lower and upper dashed lines represent the 25th and 75th quantiles, respectively. *p ≤ 0.05, **p ≤ 0.01, and ***p ≤ 0.001. CHP‐9, cyclohexapeptide‐9.

Treatment with retinol decreased the a* value significantly by −0.27 (95% CI: −0.41, −0.13; *p* < 0.001) compared to what the control did. In the meantime, the decrease of a* value by CHP‐9 treatment extended from −0.43 (95% CI: −0.67, −0.19; *p* < 0.001) on Day 14 to −1.03 (95% CI: −1.49, −0.58; *p* < 0.001) on Day 56 (Table 2). In addition, CHP‐9 treatment decreased the area of skin with erythema significantly by −3.58 mm^2^ (95% CI: −6.15, −1.01; *p* = 0.00632), while no significant reduction was introduced by retinol treatment (Table 1 and Figure S4). In accordance with the reduction in the a* value, the erythema index (EI) was also reduced by retinol to an extent of −5.97 (95% CI: −8.67, −3.27; *p* < 0.001), while the reduction by CHP‐9 treatment became more and more robust until a final value of −25.25 (95% CI: −35.34, −15.15; *p* < 0.001) on Day 56 (Table 2). Multiple comparisons of the relative changes confirmed that the relative improvements in the a* value and EI were significantly higher in the CHP‐9 group than in the control group on Days 28 and 56 (Figure 4A,B). Furthermore, the relative changes in the area of erythema were also significantly higher in the CHP‐9 group than in the retinol group across the study (Figure 4C). The parameters for skin barrier function, that is, skin hydration and trans‐epidermal water loss (TEWL), were simultaneously improved by retinol and CHP‐9 treatment both in a time dependent manner, only that the potency of CHP‐9 was higher than that of retinol. On Day 56, retinol increased the skin hydration by 4.22 (95% CI: 3.44, 5.00; *p* < 0.001) and decreased the TEWL by −1.59 g/h/m^2^ (95% CI: −1.93, −1.24; *p* < 0.001), respectively, while CHP‐9 increased the skin hydration by 9.44 (95% CI: 8.35, 10.53; *p* < 0.001) and decreased the TEWL by −3.93 g/h/m^2^ (95% CI: −4.59, −3.27; *p* < 0.001), respectively (Table 2). Multiple comparisons demonstrated that the relative changes in the skin hydration and TEWL at each visit were all significantly higher in the CHP‐9 group than in the retinol group, and the absolute value of hydration was significantly higher in the CHP‐9 group than in the retinol group on Day 56 (Figure 4D,E). In addition to the functions of the skin barrier, the structural changes in the thickness and density of the epidermis were also measured. Treatment with retinol increased the thickness and density of the epidermis by 2.00 μm (95% CI: −0.05, 4.05; *p* = 0.05582) and 1.57% (95% CI: 0.60, 2.54; *p* = 0.0015), respectively. On the other hand, CHP‐9 significantly increased the thickness and density of the epidermis in a time dependent manner to an extent of 8.87 μm (95% CI: 4.54, 13.20; *p* < 0.001) and 5.61 (95% CI: 4.00, 7.21; *p* < 0.001) on Day 56, respectively (Table 2 and Figure S5). Pairwise comparisons further revealed that the relative improvement in the epidermal density by CHP‐9 was significantly higher than that by retinol on Day 56 (Figure 4F,G).

### 3.4 Dermatological Assessment of Facial Wrinkles

**FIGURE 5:**
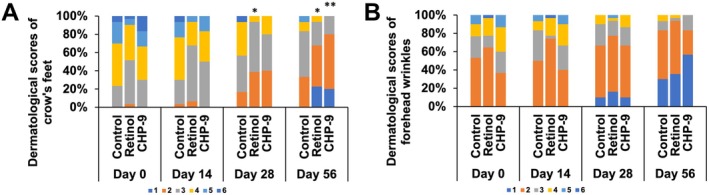
Dermatological assessment of (A) crow's feet and (B) forehead wrinkles. Crow's feet and forehead wrinkles were assessed with a 7‐point and a 9‐point scale, respectively. A higher score indicates a worse skin condition. *p ≤ 0.05 and **p ≤ 0.01 compared to the control group. CHP‐9, cyclohexapeptide‐9.

In consistent with the measured results, dermatological assessments of skin wrinkles demonstrated that both retinol and CHP‐9 could improve skin wrinkles, only that CHP‐9 was more potent than retinol. Subjects in the retinol group had estimated odds of higher dermatological scores for crow's feet 0.27778 (0.1203, 0.6452; *p* = 0.0028) times the corresponding odds in the control group (Table 3). On the other hand, subjects in the CHP‐9 group had estimated cumulative odds of unfavorable outcomes decreased continuously from 0.3390 (0.1379, 0.8333; *p* = 0.0184) times on Day 14 to 0.0244 (0.006, 0.0981; *p* < 0.001) times the odds in the control group on Day 56 (Table 3). No significant effect of retinol was noticed on the dermatological assessment of the forehead wrinkles, while CHP‐9 demonstrated a time dependent decrease in the forehead wrinkles and reached significant on Day 56 (Table 3). Multiple comparisons indicated that CHP‐9 treated group differed significantly from the control group in the composition of dermatological scores for crow's feet on Day 56, while retinol improved crow's feet on both Days 28 and 56 (Figure 5). In addition to the assessment of crow's feet and forehead wrinkles, dermatologists were also required to assess cheek wrinkles, undereye wrinkles, and nasolabial folds. CHP‐9 consistently improved wrinkling symptoms in all three areas, while retinol only exhibited beneficial effects on undereye wrinkles and nasolabial folds (Table 3 and Figure S6).

**TABLE 3:** GEE outputs for dermatologically assessed outcomes.

Outcome	Treatment^a^	OR (95% CI)	OR by time (95% CI)		
			Day 14	Day 28	Day 56
Crow's feet	Retinol	**0.28 (0.12, 0.65)** **	/	/	/
	**CHP‐9**	/	**0.34 (0.14, 0.83)** *	**0.10 (0.03, 0.35)** ***	**0.02 (0.01, 0. 10)** ***
Forehead wrinkles	Retinol	0.57 (0.28, 1.14)	/	/	/
	**CHP‐9**	/	1.12 (0.51, 2.50)	0.28 (0.07, 1.05)	**0.04 (0.01, 0.25)** ***
Cheek wrinkles	Retinol	0.49 (0.19, 1.28)	/	/	/
	CHP‐9	0.26 (0.10, 0.68)**	/	/	/
Undereye wrinkles	Retinol	0.47 (0.24, 0.92)*	/	/	/
	**CHP‐9**	/	0.70 (0.40, 1.22)	0.22 (0.05, 0.87)*	0.01 (0.002, 0.08)***
Nasolabial folds	Retinol	0.15 (0.06, 0.43)***	/	/	/
	CHP‐9	0.12 (0.04, 0.35)***	/	/	/
Dullness	Retinol	0.25 (0.11, 0.58)**	/	/	/
	CHP‐9	0.04 (0.02, 0.11)***	/	/	/
Glossiness	Retinol	0.30 (0.12, 0.73)**	/	/	/
	CHP‐9	0.09 (0.04, 0.21)***	/	/	/
Dryness	**Retinol**	/	0.23 (0.09, 0.64)**	0.21 (0.07, 0.63)**	0.05 (0.01, 0.19)***
	**CHP‐9**	/	0.04 (0.01, 0.14)***	0.01 (0.002, 0.04)***	0.003 (0.0004, 0.03)***
Erythema	Retinol	0.26 (0.13, 0.55)***	/	/	/
	CHP‐9	0.06 (0.02, 0.15)***	/	/	/
Firmness	Retinol	0.36 (0.15, 0.83)*	/	/	/
	CHP‐9	0.04 (0.01, 0.12)***	/	/	/
Evenness	Retinol	0.19 (0.08, 0.49)***	/	/	/
	**CHP‐9**	/	0.16 (0.06, 0.44)***	0.04 (0.01, 0.13)***	0.09 (0.02, 0.40)**
Roughness	**Retinol**	/	0.30 (0.12, 0.80)*	0.31 (0.11, 0.92)*	0.05 (0.01, 0.18)***
	CHP‐9	0.03 (0.01, 0.08)***	/	/	/

*Note:* Crow's feet and forehead wrinkles were assessed by dermatologists using a 7‐point scale and a 9‐point scale, respectively. Cheek wrinkles and undereye wrinkles were assessed using a 10‐point scale, while nasolabial folds were assessed with an 8‐point scale. Skin dullness, glossiness, dryness, erythema, firmness, evenness, and roughness were assessed each using a 10‐point scale. For all these scales, a higher score indicates a worse skin condition. Dermatological assessed outcomes were treated as ordinal variables and were analyzed with a GEE solver for correlated ordinal multinomial responses with the vehicle control set as the reference.

Abbreviations: CHP‐9, cyclohexapeptide‐9; GEE, generalized estimating equation.

^a^ Bold text means a significant treatment‐by‐time interaction was detected.

* *p* ≤ 0.05 compared to the vehicle control group.

** *p* ≤ 0.01 compared to the vehicle control group.

*** *p* ≤ 0.001 compared to the vehicle control group.

Other skin appearances related to skin pigmentation, firmness, and evenness, and barrier function were also evaluated by dermatologists. Basically, CHP‐9 consistently outperformed retinol in all the measurements and assessments with a larger difference from the control than retinol. Furthermore, CHP‐9 showed progressively augmented effects on all the instrumental parameters except for R7, b* value, and erythema area, though only for dermatological assessments of undereye wrinkles, dryness, and evenness was this pattern documented. In the cases of instrumentally measured F4, hydration, and TEWL, and dermatologically assessed dryness and roughness, retinol also showed time‐dependent effects, but their extents were consistently lower than in CHP‐9 (Table 3 and Figures S7–S9).

### 3.5 Tolerability

None of the treatment‐related adverse events were reported in any of the treatment groups.

## 4 Discussion

In the present study, we compared the anti‐aging effects of an innovatively cyclized collagen peptide CHP‐9 with that of retinol and found that CHP‐9 exceeded retinol in many aspects of dealing with skin aging. As a safer substitution for retinol, peptides have drawn much attention from the scientific community for anti‐aging purposes, only that their application is limited by relatively low stability, skin permeation, and target selectivity. Here, we for the first time cyclized hexapeptide‐9 by linking its carboxy and amino termini, aiming to improve its stability, skin permeability, and bioactivity. Our results not only confirmed the effects of CHP‐9, but also demonstrated that CHP‐9 was superior to retinol in reducing skin wrinkling, improving skin elasticity, reducing skin pigmentation, and repairing skin barrier function. Moreover, for most of the parameters, CHP‐9 exerted its actions in a progressively augmented manner as the time of application increased, whereas the effects of retinol remained constant during the study. In this case, even for a few parameters that CHP‐9 showed lower potency compared to that of retinol at the early stage of the trial, the effects kept enhancing and finally exceeded retinol at the late stage of the trial. Therefore, we believe that long‐term use of CHP‐9 is a practical therapeutic option for skin aging, provided its long‐term safety is secured by continuously monitoring any consumer‐reported incidence of adverse reactions in real‐world application scenarios for at least 3 years.

Retinoids consist of a cyclic six‐carbon ring and an isoprenoid side chain, which contains four all‐*trans* unsaturated double bonds that are susceptible to oxidation under the influence of diverse factors, including light, high temperature, strong acids, and oxygen [26]. Retinol degrades mainly by isomerization to cis‐isomers, molecular fragmentation, and photochemical oxidation and reduction reactions [27, 28]. In this regard, the Scientific Committee on Consumer Safety (SCCS) has emphasized the issue of a lack of stability data on retinol in cosmetic formulations and the need for proper stabilization measures in final formulations [29]. Recently, a comprehensive study on the stability and degradation kinetics of common retinoids in cosmetic formulations indicated that as much as 75% of retinol degraded after 6 months at 25°C, which was further enhanced by high temperature and light exposure [30]. On the other hand, cyclization of hexapeptide‐9 has significantly increased its stability against enzymatic hydrolysis due to both ends of the chain having been hidden from proteinase recognition. Our internal data has proven that more than 95% of CHP‐9 remained after 24 h hydrolysis by collagenase type I, whereas linear hexapeptide‐9 was completely hydrolyzed. This increased stability against enzymatic hydrolysis may have led to the accumulation of CHP‐9 in the dermis as time increased, which may explain the progressively augmented performances of CHP‐9.

Skin absorption of active substances depends on their physicochemical properties, which prefer a molecular weight ≤ 500 Da, a moderate Log *P* of 1–3.3, an aqueous solubility of more than 1 mg/mL, and no or slight polarity [31, 32]. Retinol, however, is highly lipophilic with a Log *P* of 5.65 [29], rendering the partitioning of retinol from the formulation to the lipophilic stratum corneum not favored [33]. One previous study has shown that even when the skin barrier was dampened by tape strapping, retinol could only penetrate 10–15 μm after 4.5 h, which is far from crossing the epidermis [33, 34]. This slow penetration of retinol, in addition to its instability, makes retinol present a low amount in the target site, because most is either degraded before percutaneous absorption or remains on the skin surface. In contrast, our internal data obtained by confocal Raman microscopy has clearly shown that CHP‐9 reached the dermis at 4 h after application. Therefore, combined with the enhanced stability against enzymatic hydrolysis, CHP‐9 could significantly increase the content of actives in the target site, that is, dermis, to exert its actions. A thorough investigation on the changes in physicochemical properties after cyclization, including solubility, Log *P*, molecular size, and polar surface area, that may determine its skin penetration is warranted.

In addition to the increase in the presentation of actives in the target site, the biological activity of hexapeptide‐9 may have also been strengthened by cyclization. Generally, the conformational rigidity of cyclic peptides allows enhanced binding toward target molecules, which brings better biological activity compared to their linear counterparts [35]. Dal Farra et al. first designed and synthesized hexapeptide‐9 to enhance intercellular adhesions and cell adhesion to the ECM [22]. Based on these effects, the inventors implied that hexapeptide‐9 may promote the production of ECM and the expression of integrins [22]. Our internal data has confirmed that CHP‐9 stimulates collagen synthesis significantly more potently than hexapeptide‐9. Our data also indicates that the expression of integrin β4 subunit can be increased by 6 folds by CHP‐9 compared to hexapeptide‐9. Therefore, the cyclization of hexapeptide‐9 not only increased its presentation in the target site, but also improved its potency against skin aging. To put the anti‐wrinkle potency of CHP‐9 in a bioactive peptides context, topical application of 3 ppm palmitoyl pentapeptide‐4 for 12 weeks only reduced fine lines/wrinkles to a small extent [36], while 100 ppm tetrapeptide GEKG for 8 weeks reduced wrinkle volume by 12.2% [37]. Thus, the anti‐wrinkle potency of CHP‐9 is relatively high given that 20 ppm of CHP‐9 decreased wrinkle number by more than 12% at 8 weeks. Future studies on head‐to‐head comparison of these bioactive peptides with anti‐wrinkle properties are warranted.

Retinol is well known for its irritating potential when used topically. However, in the current study, no irritating event was reported by participants or investigators. This absence of irritating side effects may be attributed to our treatment strategy, which was to minimize side effects to permit daily use over a long period. In the present study, the content of retinol in the serum was only 0.002% (*w*/*w*), which is the minimal content required in cosmetic products (0.0015%–0.3%) [29]. This under‐dosing of retinol may be the major reason for the lack of irritation events in our study. In a more common practice, 0.1% retinol has recently been shown to significantly increase the incidence of skin erythema compared to vehicle control [38]. Our study also showed a noticeable placebo effect in the vehicle group. Depending on the parameter, this may be at least in part attributed to the moisturizing activity of the vehicle serum. This may have also helped to reduce the irritating potential of the retinol serum.

In the present study, instrumental measurement was combined with dermatological assessment to strengthen the accuracy and reliability of our results, with dermatological assessment as a supplement to instrumental measurement. Currently, clinical features remain the gold standard for diagnosing various skin diseases and conditions in clinical practices. However, the diverse nature of these skin conditions and the presence of various subjective scales with no validation make an objective and precise assessment challenging [39, 40]. Thus, a wide range of noninvasive evaluation techniques have been employed by dermatologists to ensure accurate assessments of skin conditions. However, researchers have also raised concerns about the reliability and validity of noninvasive instrument measurements because they are susceptible to environmental influences [41, 42]. A recent study systemically reviewed studies with device measurements and confirmed that the most reliable device to evaluate skin texture in ordinary skin was PRIMOS^CR^, which was the same model used to measure wrinkles in our present study [43]. In addition, our results of dermatological assessment aligned well with those of the instrumental measurements of wrinkles. Together, both instrumental measurements and dermatological assessments suggested that CHP‐9 not only is more potent than retinol in improving skin wrinkles but also works in a pattern of progressive augmentation.

Our study has some limitations. Firstly, no adverse event was noticed for any of the treatments, which did not reflect the superior safety profile of CHP‐9 over retinol. Secondly, the participants were not instructed to use UV protection during the study to exclude the potential impacts of different sun exposure among participants on their skin conditions. Lastly, we did not evaluate the protective effects of the treatments on the neck region, which is considered to be one of the most susceptible zones to photoaging.

## 5 Conclusions

In summary, our preliminary data indicates that cyclized hexapeptide‐9 may have provided a promising alternative to retinol for protecting against skin aging. Given the hyper‐safety profile of peptides, topical use of CHP‐9 may present a better option for skin aging prevention/therapeutics. Further studies are warranted to confirm the superiority of cyclized hexapeptide‐9 over retinol in larger populations.

## Author Contributions

Conceptualization: Huailong Chang, Shengnan Tang, and Haining Yu. Methodology: Huailong Chang, Kan Tao, Yanling Wang, and Yuge Yang. Investigation: Huailong Chang, Kan Tao, Xiaoli Wang, and Mengru Ge. Data curation: Huailong Chang, Yuge Yang, and Mengru Ge. Writing – original draft preparation: Huailong Chang. Writing – review and editing: Shengnan Tang and Haining Yu. Visualization: Huailong Chang and Yuge Yang. Supervision, Shengnan Tang and Haining Yu.

## Ethics Statement

Written informed consent was obtained from each patient after the procedures, risks, and benefits had been explained. The trial protocol was approved by the Institutional Review Board and Ethics Committee in the study center (Approval No.: 2024‐QX‐05‐02). The trial was conducted in accordance with the principles of the Declaration of Helsinki and Good Clinical Practice guidelines of the International Council for Harmonization.

## Conflicts of Interest

The authors declare no conflicts of interest.

## Supporting information


Data S1.

## Data Availability

The data that support the findings of this study are available on request from the corresponding author. The data are not publicly available due to privacy or ethical restrictions.
