# Supplementary material for: Novel Cyclized Hexapeptide‐9 Outperforms Retinol Against Skin Aging: A Randomized, Double‐Blinded, Active‐ and Vehicle‐Controlled Clinical Trial
Source: J Cosmet Dermatol. 2025 Jun 30;24(7):e70290. doi: 10.1111/jocd.70290 (PMC12207714; doi:10.1111/jocd.70290)
Supplement: Supplementary file 1 — Data S1. [file JOCD-24-e70290-s001.docx]

**Supplementary Materials**

**Supplementary Table 1.** Tested formulations.

| **Trade name/Supplier** | **INCI** | **%, w/w** | | |
| --- | --- | --- | --- | --- |
|  |  | **Control** | **VA** | **CHP-9** |
| *Phase A* |  |  |  |  |
| Purified Water | WATER | To 100 | To 100 | To 100 |
| Dissolvine Na2/Nouryon | DISODIUM EDTA | 0.0300 | 0.0300 | 0.0300 |
| PEMULEN™ TR-2 Polymer/Lubrizol | ACRYLATES/C10-30 ALKYL ACRYLATE CROSSPOLYMER | 0.1000 | 0.1000 | 0.1000 |
| Hydrolite® 8/Symrise | CAPRYLYL GLYCOL | 0.0500 | 0.0500 | 0.0500 |
| Allantoin/Shuangyou | ALLANTOIN | 0.1000 | 0.1000 | 0.1000 |
| *Phase B* |  |  |  |  |
| Aminol DHG/eleco | DIOCTYLDODECYL LAUROYL GLUTAMATE | 0.1000 | 0.1000 | 0.1000 |
| SOLUBILISANT LRI/Sensient | PPG-26-BUTETH-26, PEG-40 HYDROGENATED CASTOR OIL, WATER | 0.1500 | 0.1500 | 0.1500 |
| Cosphaderm® SB-75H/Cosphatec | HYDROGENATED LECITHIN | 0.4000 | 0.4000 | 0.4000 |
| TECKNO GLYCERIN 995/Teck Guan | GLYCERIN | 3.0000 | 3.0000 | 3.0000 |
| 1,3-BUTYLENE GLYCOL-P/KH Neochem | BUTYLENE GLYCOL | 2.0000 | 2.0000 | 2.0000 |
| Paester™ COCC/Patech | COCO-CAPRYLATE/CAPRATE | 1.2997 | 1.2997 | 1.2997 |
| Paester™ TDTM/Patech | TRIDECYL TRIMELLITATE | 0.5000 | 0.5000 | 0.5000 |
| *Phase C* |  |  |  |  |
| Aristoflex AVC/Clariant | AMMONIUM ACRYLOYLDIMETHYLTAURATE/VP COPOLYMER | 0.3600 | 0.3600 | 0.3600 |
| Dipropylene Glycol LO+/DOW | DIPROPYLENE GLYCOL | 4.0000 | 4.0000 | 4.0000 |
| *Phase D* |  |  |  |  |
| L-Arginine/Kyowa | ARGININE | 0.0800 | 0.0800 | 0.0800 |
| Purified Water | WATER | 0.7200 | 0.7200 | 0.7200 |
| *Phase E* |  |  |  |  |
| Purified Water | WATER | 1.0000 | 1.0000 | 1.0000 |
| 1,3-BUTYLENE GLYCOL-P/KH Neochem | BUTYLENE GLYCOL | 1.0000 | 1.0000 | 1.0000 |
| SymSave® H (672096)/Symrise | HYDROXYACETOPHENONE | 0.5000 | 0.5000 | 0.5000 |
| *Phase F* |  |  |  |  |
| PURITY 21C PURE/Nouryon | ZEA MAYS (CORN) STARCH | 0.3000 | 0.3000 | 0.3000 |
| DRY-FLO PURE/Nouryon | ALUMINUM STARCH OCTENYLSUCCINATE | 0.3000 | 0.3000 | 0.3000 |
| 1,3-BUTYLENE GLYCOL-P/KH Neochem | BUTYLENE GLYCOL | 2.0000 | 2.0000 | 2.0000 |
| *Phase G* |  |  |  |  |
| effisin™ hd multifunctional/Ashland | 1,2-HEXANEDIOL | 0.6500 | 0.6500 | 0.6500 |
| sensiva™ sc 50 multifunctional/Ashland | ETHYLHEXYLGLYCERIN, TOCOPHEROL | 0.0500 | 0.0500 | 0.0500 |
| Anallerg® Pure-origin® (Retinol)/Coachchem | RETINOL | 0.0000 | 0.0020 | 0.0000 |
| Cyclohexapeptide-9 |  | 0.0000 | 0.0000 | 0.0020 |

INCI, international nomenclature for cosmetic ingredients; CHP-9, cyclohexapeptide-9.

**Supplementary Figure 1.** CONSORT Flow diagram of participant disposition.


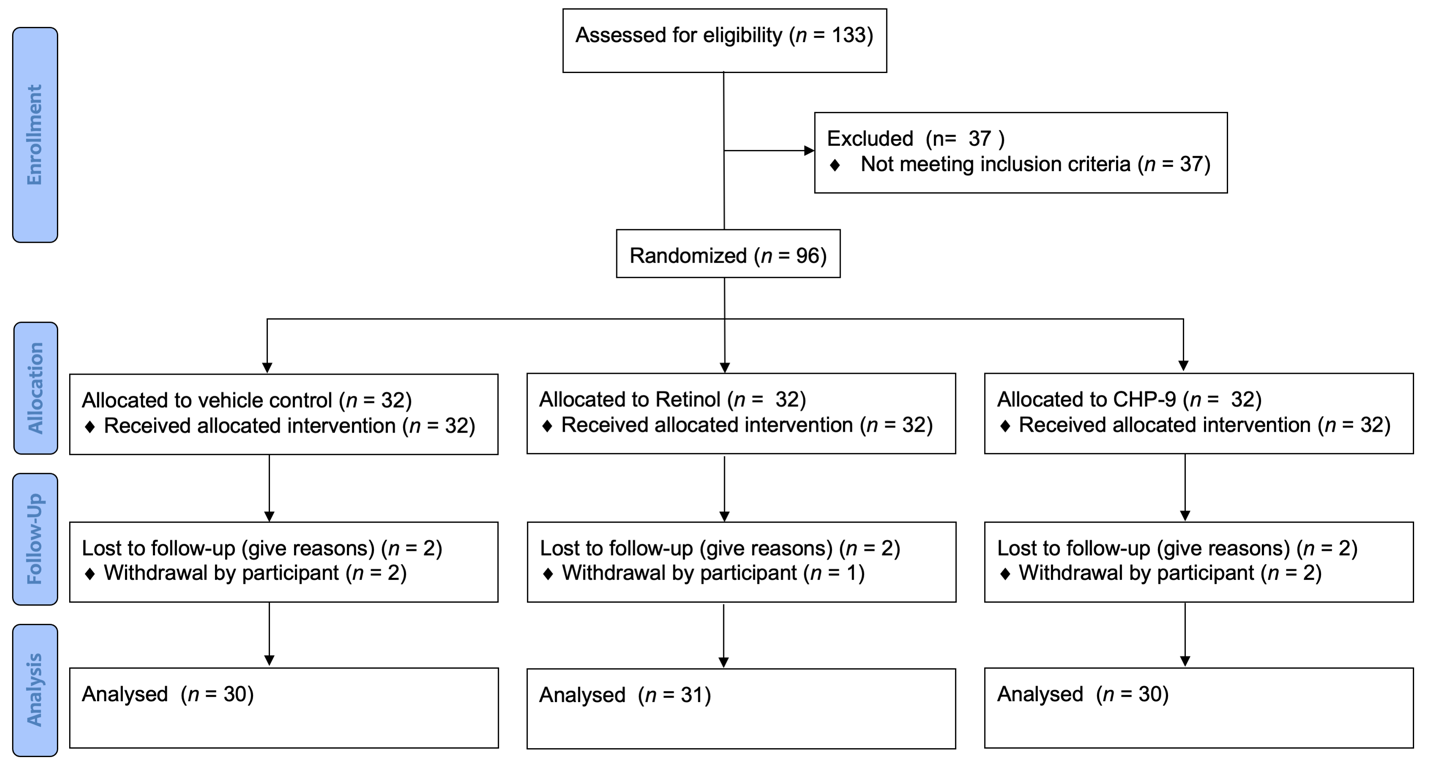


**Supplementary Figure 2.** Representative images of crow’s feet taken by a PRIMOS^CR^ system.


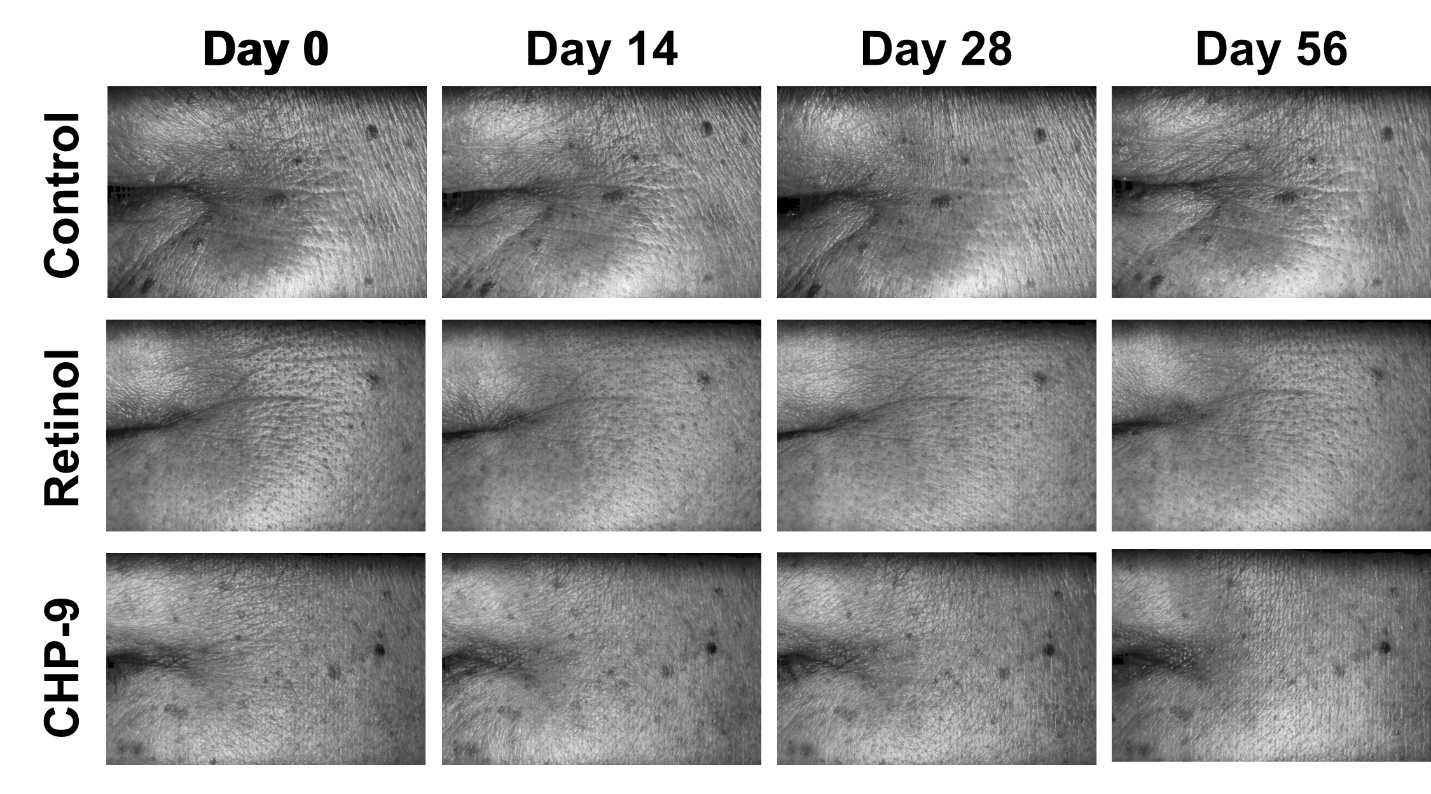


**Supplementary Figure 3.** Representative images of forehead wrinkles taken by a PRIMOS^CR^ system.


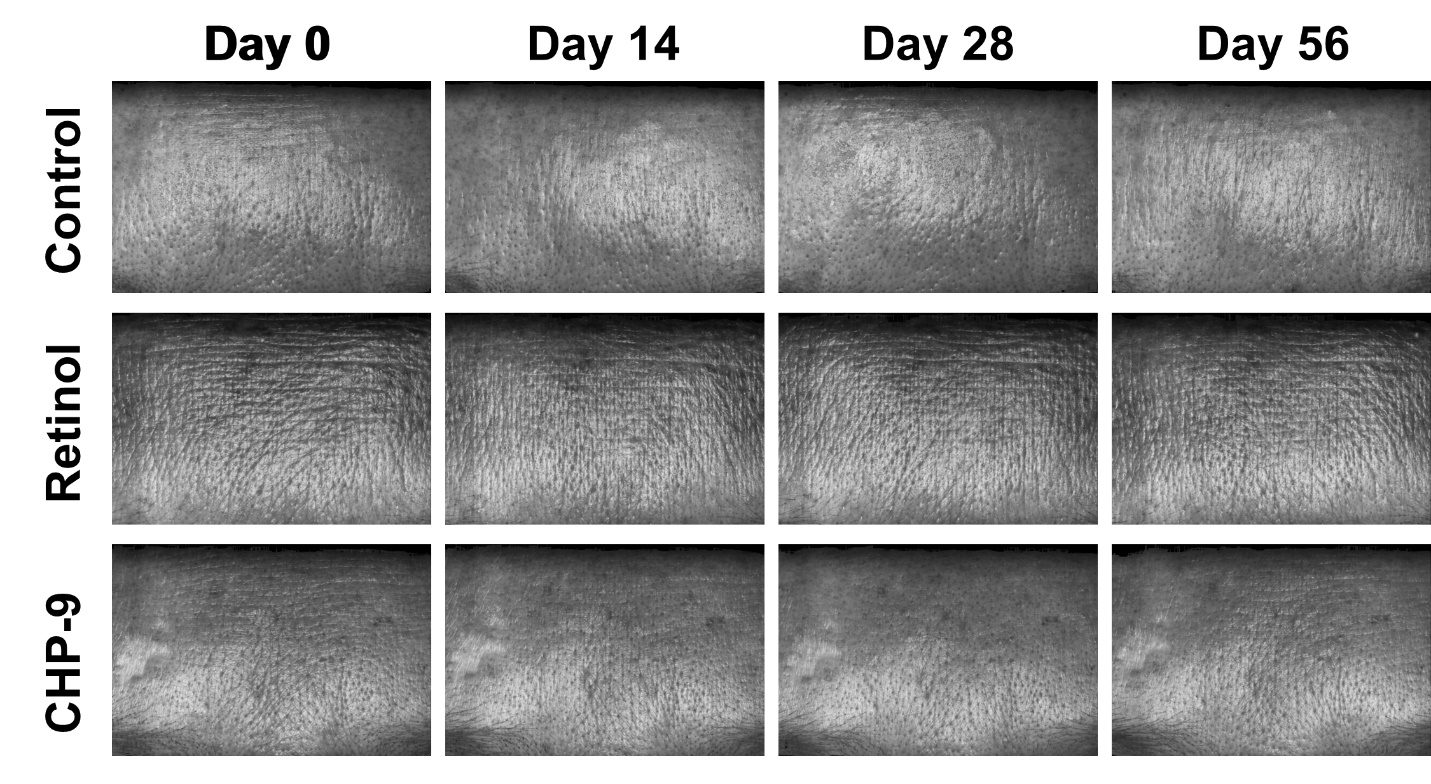


**Supplementary Figure 4.** Representative images of skin erythema taken by a VISIA®-CR system.


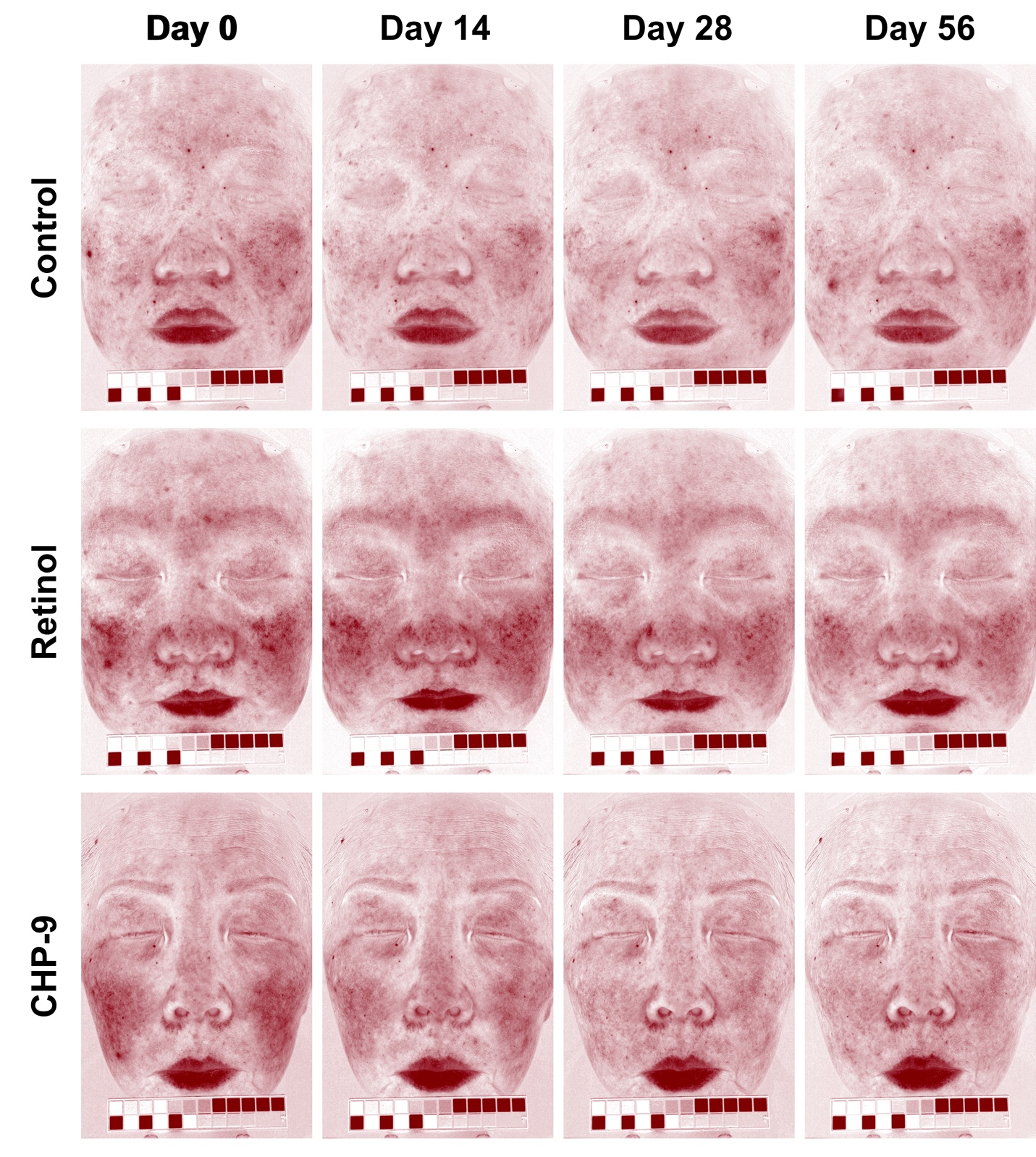


**Supplementary Figure 5.** Representative images of the thickness and density of epidermis taken by Ultrascan UC 22 probe connected to the Cutometer® dual MPA 580 system.


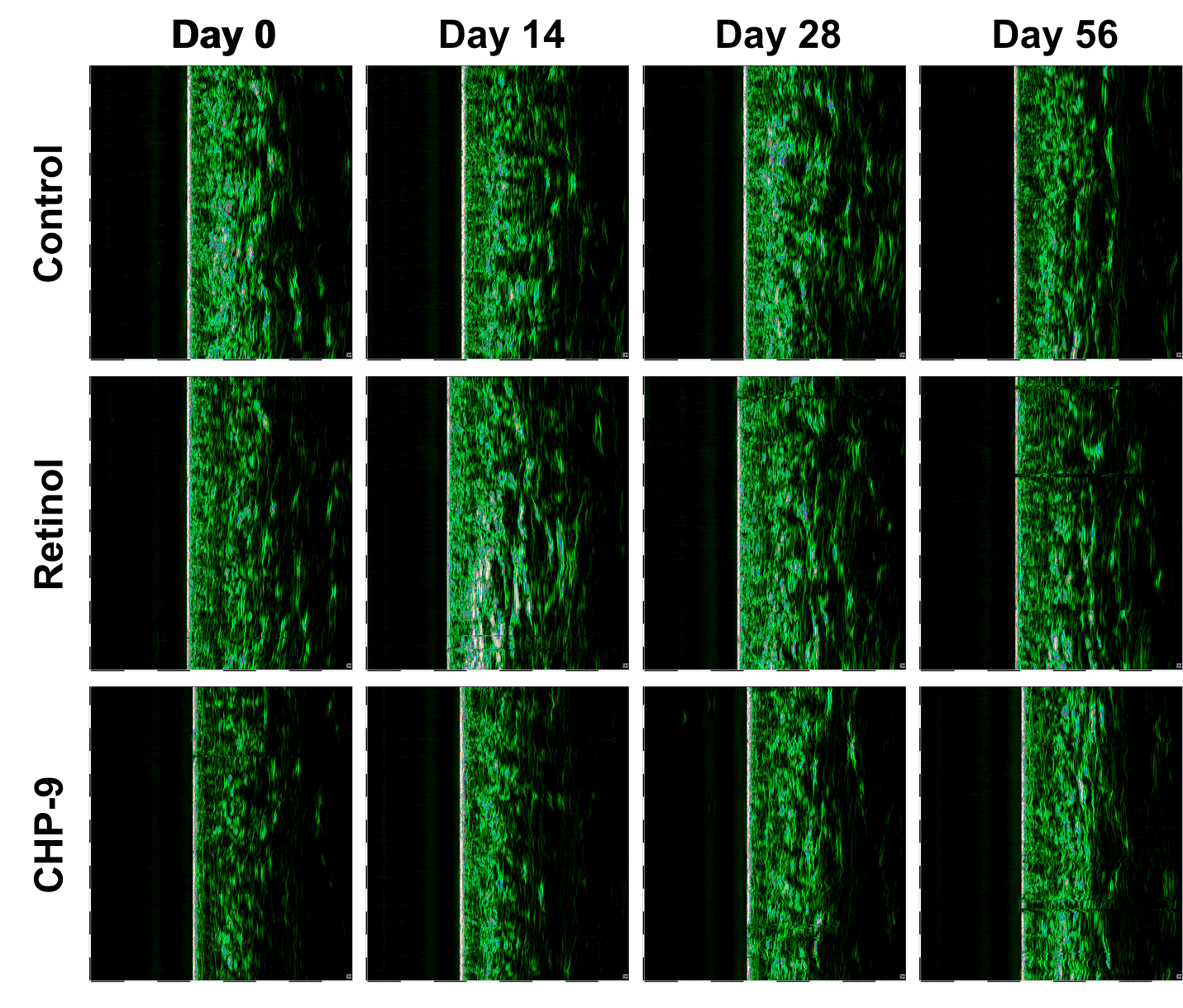


**Supplementary Figure 6.** Dermatological assessment of cheek wrinkles, undereye wrinkles, and nasolabial folds.


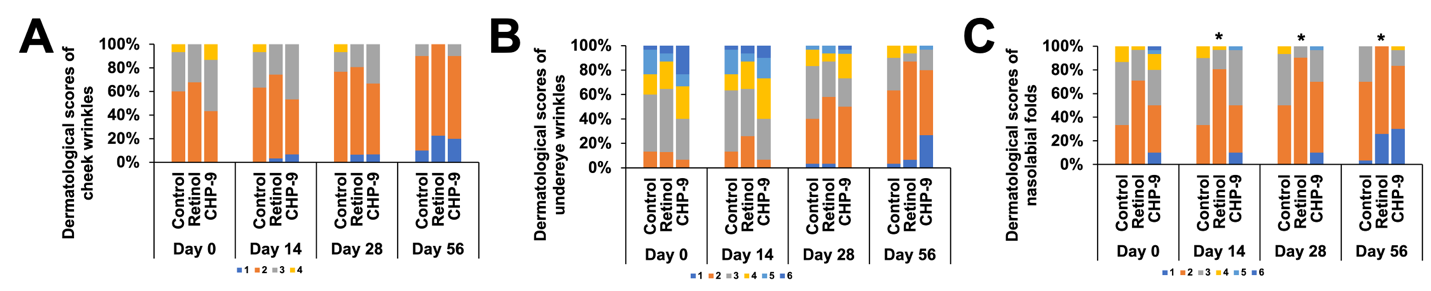


Cheek wrinkles and undereye wrinkles were assessed using a 10-point scale, while nasolabial folds were assessed with an 8-point scale by comparing to guideline photos. For all these scales, a higher score indicates a worse skin condition. Multiple comparisons were performed using a resampling-based method to adjust the *P* values to account for type I error rate. **P* ≤ 0.05 compared to the vehicle control group. CHP-9, cyclohexapeptide-9.

**Supplementary Figure 7.** Dermatological assessment of skin elasticity and firmness.


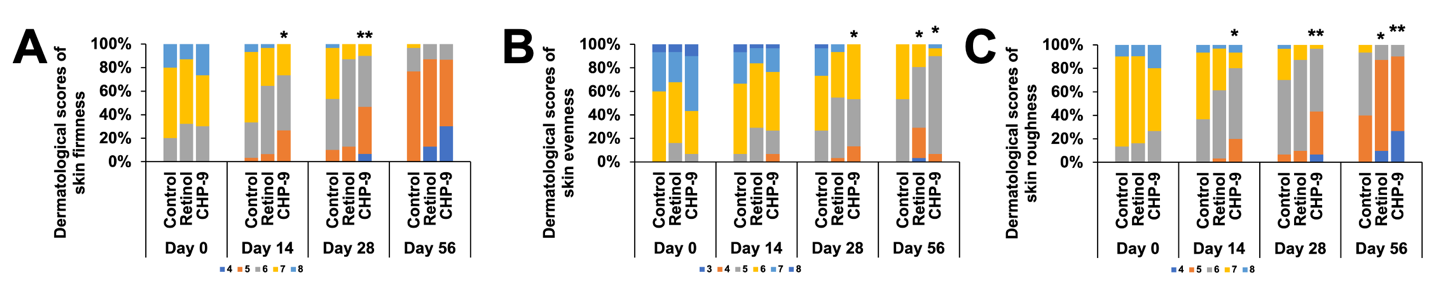


Skin firmness, evenness, and roughness were assessed each using a 10-point scale by comparing to guideline photos. For all these scales, a higher score indicates a worse skin condition. Multiple comparisons were performed using a resampling-based method to adjust the *P* values to account for type I error rate. **P* ≤ 0.05 and ***P* ≤ 0.01 compared to the vehicle control group. CHP-9, cyclohexapeptide-9.

**Supplementary Figure 8.** Dermatological assessment of skin tones.


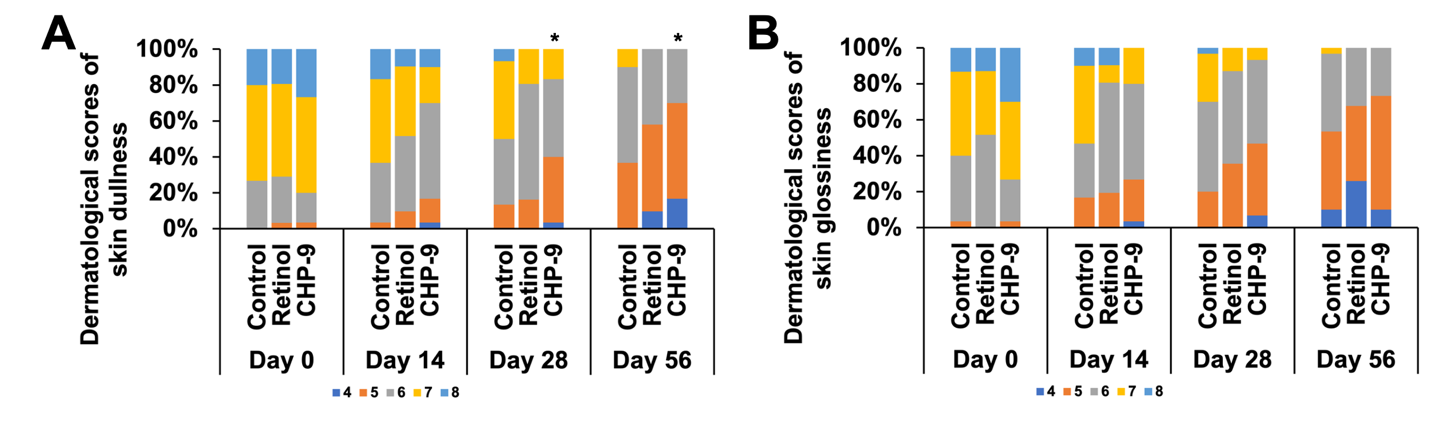


Skin dullness and glossiness were assessed each using a 10-point scale by comparing to guideline photos. For all these scales, a higher score indicates a worse skin condition. Multiple comparisons were performed using a resampling-based method to adjust the *P* values to account for type I error rate. **P* ≤ 0.05 compared to the vehicle control group. CHP-9, cyclohexapeptide-9.

**Supplementary Figure 9.** Dermatological assessment of skin barrier function.


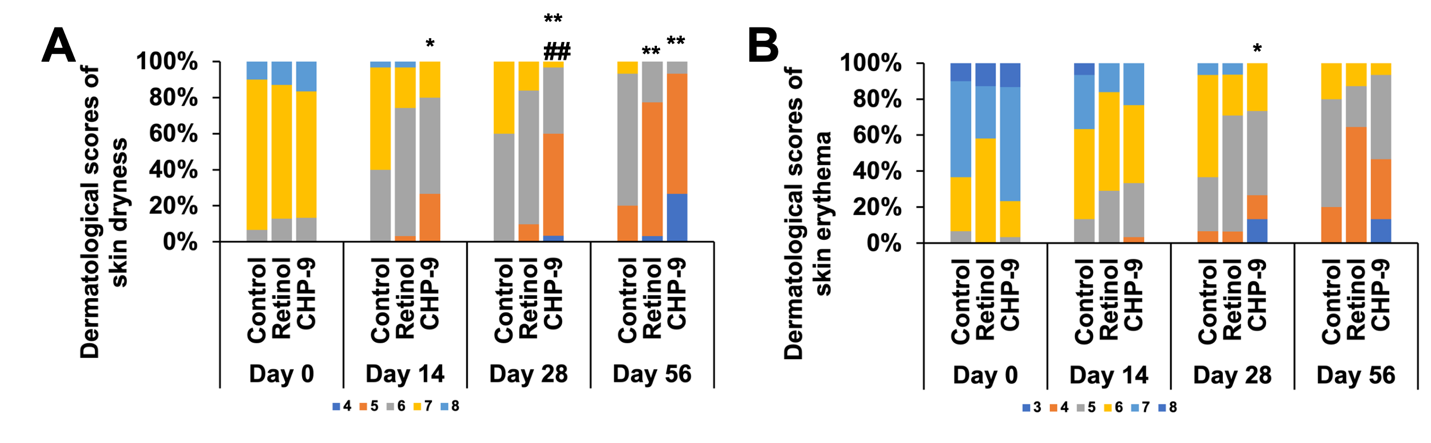


Skin dryness and erythema were assessed each using a 10-point scale by comparing to guideline photos. For all these scales, a higher score indicates a worse skin condition. Multiple comparisons were performed using a resampling-based method to adjust the *P* values to account for type I error rate. **P* ≤ 0.05 and ***P* ≤ 0.01compared to the vehicle control group; ^##^ *P* ≤ 0.01 compared to the retinol group. CHP-9, cyclohexapeptide-9.
